# Editorial: Control of Pestivirus Infections in the Management of Wildlife Populations

**DOI:** 10.3389/fmicb.2016.01396

**Published:** 2016-09-08

**Authors:** Julia F. Ridpath, Thomas Passler

**Affiliations:** ^1^Ruminant Diseases and Immunology Research Unit, National Animal Disease Center, Agricultural Research Service-United States Department of AgricultureAmes, IA, USA; ^2^Department of Clinical Sciences, College of Veterinary Medicine, Auburn UniversityAuburn, AL, USA

**Keywords:** bovine viral diarrhea virus, border disease virus, classical swine fever virus, emerging, pestivirus, wildlife

The genus pestivirus in the Flavivirus family is comprised of single-stranded RNA viruses that infect domestic and wildlife hosts. The “classic” pestivirus species, bovine viral diarrhea virus type 1 (BVDV1), bovine viral diarrhea virus type 2 (BVDV2), border disease virus (BDV), and classical swine fever virus (CSFV) were first detected in domestic animals and early differentiation of pestivirus species was based on the domestic animal from which it was isolated. However, it became apparent that these four species were capable of infecting multiple domestic and free-ranging host species. In addition, emerging/putative species of pestivirus, such as pronghorn virus and giraffe virus, were isolated exclusively from wildlife species. The significant economic cost of the infection of domestic species with pestiviruses is well documented but the impact of pestivirus infections on wildlife species is less well studied.

Early reports of the isolation of pestiviruses from wildlife were treated as incidental findings. As eradication efforts for CSFV, BVDV1, and BVDV2 proceed around the world, concerns have been raised that wildlife species may act as reservoirs for pestiviruses. Data indicate that CSFV-infected Eurasian wild boar (*Sus scrofa*) pose a considerable threat to eradication programs in domestic swine. This has led to the implementation of CSFV control strategies in wild boar that combine detection, culling (with cooperation of hunters), and vaccination as detailed in the chapter by Moennig in this volume. Additionally, the chapter by Rossi et al. details the design and use of oral vaccines in wild boar populations.

The possibility of free-ranging cervids, small wild ruminants, and rabbits to be reservoirs for BVDV1 and BVDV2 is less clear. Viral detection and serological surveillance both indicate that multiple species of wild ruminants are susceptible to BVDV1 and BVDV2 infection as reported in the chapter by Wolff et al. Epidemiological data indicate that in North America, BVDV1 and BVDV2 infections have become established in wild ruminant populations, and that persistently infected animals are present in these populations as described for white-tailed deer in the chapter by Passler et al. However, to date, evidence demonstrating the introduction of pestiviruses into naïve cattle herds by exposure to wildlife is limited. In Europe, several free-ranging species have been investigated for the potential to be reservoir hosts for BVDV, and the chapters by Larska and Grant et al. discuss the potential for reindeer and rabbits as vectors for the reintroduction of BVDV1 into naive cattle herds. While questions regarding the role of wildlife species as reservoirs remain, it is becoming increasingly apparent that pestiviruses pose a threat to the health of captive and free-ranging wildlife.

The potential of pestivirus infections to endanger wildlife species, such as chamois and big horn sheep should not be underestimated. The impact of BDV infections on reintroduced Pyrenean chamois (*Rupicapra pyrenaica pyrenaica*) demonstrate that pestivirus infections can contribute to the extinction of wild populations as detailed in the chapter by Serrano et al. While the population impact is less well understood, there is clear evidence for widespread circulation of BVDV in Rocky Mountain bighorn sheep (*Ovis canadensis, canadensis*), mountain goats (*Oreamnos americanum*), and mule deer (*Odocoileus hemionus*), and BVDV infection may contribute to pneumonia die-offs or reproductive disease as discussed by Wolff et al.

Captive wildlife species in zoological collections exist in a unique environment for disease transmission and circulation of pestiviruses in zoo and parks has been demonstrated as described in the chapter by Kottwitz and Ortiz. Among pestiviruses, BVDV 1 and 2 are of special concern in zoos due to the numerous exotic ruminant species these viruses can infect. As in cattle, persistently infected animals are central in the epidemiology of BVDV and have been described in many captive and free-ranging species, including captive mountain goats, as described by Nelson et al. An additional source for the introduction of BVDV into susceptible populations is embryo transfer, which is increasingly used in the propagation of at-risk wildlife populations. Therefore, it is important that screening of all reagents, particularly fetal bovine serum, for pestiviruses become a part of standard protocols.

The chapter by Ridpath and Neill discusses the challenges in detecting and determining the impact of pestivirus infections in wild populations. The surveillance for pestivirus infections in free-ranging wildlife species is hampered by the lack of species-specific reagents. In addition, surveillance of free-ranging species is hindered by the lack of regular surveillance programs and challenges in collective representative samples from wild populations. Estimation of the impact of pestivirus infections on population health rests on the development and institution of regular surveillance programs that focus on free-ranging and captive wildlife. Such programs will be highly dependent on a local veterinary support working closely with hunters, wildlife, and forestry agencies.

Two of the emerging pestivirus species, pronghorn virus and giraffe virus, have only been detected in wildlife species. The first detection of both species was incidental. It is probable that an organized surveillance of free ranging ruminants, particularly in the African continent, would detect other pestivirus species. Detection would, once again, be dependent on research reagents specific to wild ruminant species and representative samples of wild populations. The emergence of detection protocols based on techniques such as next generation sequencing may to some extent lessen the need for species specific reagents. However, the procurement of representative samples will remain a challenge.

The lack of host-specificity allow pestiviruses to infect domestic livestock as well as captive and free-ranging wildlife, posing unique challenges to different stakeholders. While current control measures for BVDV are focused only on cattle, increased attention on the status wildlife species is necessary, as is already done for CSFV control. The impact of pestiviruses on captive and free-ranging wildlife is less well understood; however, examples of substantial damage exist, necessitating increased research attention on the effects of pestiviruses on the health of heterologous hosts. As noted above, it is probable that there exist other pestivirus species in wildlife populations. Therefore, another research focus should be on the development and implementation of appropriate tools that allow detection of these novel pestiviruses. Although great advances have been made over the last decades, novel discoveries, such as the expansive role of heterologous hosts in the epidemiology of pestiviruses, continue to shape the understanding of the “classic” pestiviruses and require further vigorous research.

## Author contributions

JR, TP contributed to the preparation, review, and revision of the manuscript.

### Conflict of interest statement

The authors declare that the research was conducted in the absence of any commercial or financial relationships that could be construed as a potential conflict of interest.

